# 6FDA-Based Co-Polyimide Membranes Incorporating Modulated MOF-808s for Olefin/Paraffin Gas Separations

**DOI:** 10.3390/membranes15100290

**Published:** 2025-09-25

**Authors:** Harun Kulak, Lore Hannes, Ivo F. J. Vankelecom

**Affiliations:** Membrane Technology Group (MTG), Centre for Membrane Separations, Adsorption, Catalysis and Spectroscopy for Sustainable Solutions (cMACS), Faculty of Bioscience Engineering, KU Leuven, Celestijnenlaan 200F, P.O. Box 2454, 3001 Leuven, Belgium

**Keywords:** mixed-matrix membranes, polyimide, metal-organic frameworks, MOF-808, gas separation, olefin/paraffin separation

## Abstract

MOF-808 was synthesized using different (perfluoro)carboxylic acid modulators, including acetic acid (AA), butyric acid (BA), trifluoroacetic acid (TFAA) and heptafluorobutyric acid (HFBA). These samples were incorporated into co-polyimide 6FDA-DAM:DABA (6FDD), and the performance of the resulting MMMs was assessed for C_2_ and C_3_ olefin/paraffin separation. Enhanced permeability was observed for both C_2_H_4_/C_2_H_6_ and C_3_H_6_/C_3_H_8_ mixtures thanks to the introduced porosity upon filler incorporation in all cases. Due to the large pore size of MOF-808, diffusion-selective permeation through the polymer phase of the MMMs determined the eventual selectivity for C2 gases, leading to separation factors similar to that of the unfilled 6FDD membrane. For C_3_H_6_/C_3_H_8_ separation, the incorporation of fluorinated MOFs significantly improved separation performance, unlike their non-fluorinated counterparts. The unfilled 6FDD membrane exhibited a C_3_H_6_/C_3_H_8_ separation factor of 7.4 with a C_3_H_6_ permeability of 22 Barrer, while the incorporation of MOF-808-TFAA and MOF-808-HFBA led to C_3_H_6_/C_3_H_8_ separation factors of 13.1 and 13.5 with corresponding improved C_3_H_6_ permeabilities of 42 Barrer and 33 Barrer, respectively. Considering that these MMMs showed C_3_H_6_ permeabilities similar to those of MMMs containing their non-fluorinated MOF counterparts that exhibited no enhancement in membrane selectivity, the improved C_3_H_6_/C_3_H_8_ separation factor was attributed to the preferential adsorption of C_3_H_8_ over C3H6 on the fluorinated MOFs, acting as a trap for C_3_H_8_ and reducing its diffusivity. These results highlight the significance of matching the permeation characteristics of the selected polymer-filler pair on MMM performance for different gas pairs.

## 1. Introduction

Ethylene and propylene play a pivotal role in the chemical industry, serving as building blocks for myriad products widely used in a variety of applications [1,2,3]. Steam cracking of saturated hydrocarbons derived from natural gas and petroleum (e.g., naphtha and liquefied petroleum gas) is the most commonly employed technology for the production of these C_2_ and C_3_ olefins [3,4,5]. However, this production strategy involves a critical step to separate these unsaturated hydrocarbons from their saturated counterparts (i.e., ethane and propane), which are inevitable components of the product streams. The subtle differences in molecular sizes, volatilities and condensabilities of these unsaturated and saturated hydrocarbons with the same carbon number aggravate the separation of these fractions to obtain high-purity olefins [6,7]. The most prevalent approach to separate these olefin/paraffin mixtures is cryogenic distillation, which operates energy-intensively and demands high capital investments [6,7,8,9]. Given the huge energy consumption and associated costs, alternative technologies to distillation, including absorption, adsorption and membrane processes, have attracted a substantial interest for olefin/paraffin separation [6,7,8,9,10,11,12,13].

Membrane technology holds great potential for gas separation thanks to its inherent advantages, such as high energy efficiency, low operating costs, small carbon footprint, and reduced need for moving parts and harmful chemicals [14,15,16]. Nevertheless, the performance of conventional polymeric membranes is limited in providing high membrane selectivity and permeability simultaneously. Among these, 6FDA-based polyimides (PIs) present one of the most promising olefin/paraffin separation performances achieved by polymeric membranes [17,18,19,20,21,22]. Besides their good thermal and chemical stability, the rigid pendant groups in the polymer structure of 6FDA-based membranes leverage the diffusion selectivity for relatively smaller olefins over paraffins due to disrupted chain packing [20,21,22]. However, the well-known trade-off between olefin/paraffin selectivity and olefin permeability still exists for these membranes.

In order to improve the permeation characteristics of polymeric membranes (i.e., enhancing selectivity, increasing permeability or ideally improving both simultaneously), mixed-matrix membranes (MMMs) have been proposed, in which filler particles are incorporated into the polymer matrix [16,17,18,23,24,25]. A wide variety of porous (nano)materials, such as zeolites, covalent-organic frameworks (COFs) and metal-organic frameworks (MOFs), has been considered as fillers for the development of such hybrid membranes [26,27,28,29]. These porous fillers can exhibit greater affinity for one gas species over another and/or provide additional diffusion pathways, altering overall gas permeation through the membrane. Upon incorporation of these fillers, MMMs can achieve outstanding separation performance, while maintaining the easy processability and mechanical strength properties exhibited by polymeric membranes [26,30,31].

MOFs are hybrid materials formed by linking inorganic metal nodes with organic ligands [32]. The versatile combination of metal centers and linker compounds has led to the discovery of numerous MOFs with diverse pore structures, exhibiting high surface areas and large pore volumes, making them major contenders for research into various gas separation applications [33]. Herein, zirconium-based MOF-808 was exploited as a porous filler considering its unique structure amenable to modification and fine-tuning, along with its high thermal stability and one-step water-based synthesis [34,35,36]. The non-structural sites of MOF-808 allow for the introduction of different functionalities through the use of different modulators and tailoring of preferential adsorption sites and pore characteristics [34].

In this study, MOF-808 samples modulated with different chain length (perfluoro)alkyl carboxylic acids were incorporated as fillers into 6FDA-DAM:DABA co-polyimide membranes to investigate their potential for challenging C_2_ and C_3_ olefin/paraffin separation. The corresponding MMMs showed improved gas permeability for both C_2_H_4_/C_2_H_6_ and C_3_H_6_/C_3_H_8_ mixtures. Beside the improved permeability, MMMs containing MOF-808 samples modulated with perfluoroalkyl carboxylic acids led to a significant enhancement in C_3_H_6_/C_3_H_8_ separation factors, highlighting the crucial role of filler-polymer pairing on the performance of membranes for specific gas separation.

## 2. Materials and Methods

### 2.1. Materials

Co-polyimide 6FDA-DAM:DABA (9:1, 6FDD) was kindly provided by Fujifilm Manufacturing Europe B.V. (Tilburg, the Netherlands) and dried at 100 °C before use. Chloroform (CHCl_3_, >99%) and acetone (Ace, >99.8%) were supplied by Acros Organics. The reagents used for the synthesis of MOF-808 samples, including zirconyl chloride octahydrate (ZrOCl_2_·8H_2_O, 98%), acetic acid (AA, ≥99.7%), butyric acid (BA, ≥99%), trifluoroacetic acid (TFAA, ≥99%) and heptafluorobutyric acid (HFBA, 98%), were purchased from Sigma-Aldrich, while 1,3,5-benzenetricarboxylic acid (BTC, 99%) was supplied by J&K Scientific. The chemical structures of 6FDD and different modulators are provided in Figure S1. Ethylene (C_2_H_4_, >99.5%), ethane (C_2_H_6_, >99.995%), propylene (C_3_H_6_, >99.5%) and propane (C_3_H_8_, >99.5%) gases were purchased from Air Liquide (Herenthout, Belgium).

### 2.2. MOF Synthesis

MOF-808 samples were synthesized using different modulators (AA, BA, TFAA or HFBA), described elsewhere [34]. Typically, a reaction mixture was prepared using 1.815 mmol of ZrOCl_2_·8H_2_O and 0.605 mmol of BTC linker in 4.55 mL of deionized (DI) water, subsequently adding 17.8 mmol of the respective modulator. The final mixture was placed in a heating block at 100 °C for 24 h under constant stirring at 100 rpm. The MOF products formed were collected by centrifugation (10 min at 4500 rpm), then washed sequentially with DI water and acetone three times. The resulting MOF-808 samples were dried in a vacuum oven at 120 °C for 16 h.

### 2.3. Membrane Preparation

For unfilled 6FDD membranes, polymer solutions were prepared by dissolving 0.2 g 6FDD in 3.8 g CHCl_3_. MMMs containing 30 wt.% filler were prepared based on an optimized recipe for the preparation of MOF-808/6FDD MMMs in our previous study [37]. First, 0.086 g MOF-808 samples were dispersed in 3.8 g CHCl_3_ using an ultrasonic bath for 15 min, and then 0.2 g 6FDD co-polyimide was added into this dispersion solution. Both unfilled and MOF-808-incorporated membranes were prepared by casting the solutions into 6 cm diameter Teflon Petri dishes under an inert nitrogen atmosphere. A funnel with a volume of 200 mL and an outlet diameter of 1 mm was placed inverted over each Petri dish to slow down the evaporation of the solvent from the cast solutions. After complete solvent evaporation and membrane solidification, the membranes were cut into coupons with a diameter of 20 mm and thermally annealed at 180 °C. For temperature increase, a heating rate of 5 °C/min was applied with an isothermal treatment at 100 °C for 1 h. The thickness of the membranes was determined by averaging the thickness values measured with a digital micrometer (Mitutoyo IP65) at five different points for each coupon.

### 2.4. Characterization of MOF and Membrane Samples

Morphologies of MOF samples and membrane cross-sections were examined with a scanning electron microscope (SEM, JEOL JSM-6010LV, Zaventem, Belgium) after sputter coating with a gold/palladium (60/40) layer (JEOL JFC-1300 auto fine coater, Zaventem, Belgium). The textural properties of MOF-808 samples were studied using a gas adsorption analyzer (Micromeritics 3Flex, Brussels, Belgium). The MOF samples were first activated under vacuum at 120 °C for 16 h using a degassing station (Micromeritics VacPrep 061, Brussels, Belgium), and then N_2_ sorption isotherms of each sample were collected at −196 °C. Surface area of the MOF-808 samples prepared with different modulators were determined according to the Brunauer–Emmett–Teller (BET) equation and pore size distributions were estimated using the nonlocal density functional theory (NLDFT) method. Crystallinity of the samples was assessed using an X-ray diffractometer (XRD, Malvern PANalytical Empyrean, Brussels, Belgium). XRD patterns were collected in the 2θ range between 1.3° and 45° using a PIXcel3D solid-state detector and X-rays generated by a Cu anode (Cu Kα_1_: λ = 1.5406 Å; Cu Kα_2_: λ = 1.5444 Å). For evaluation of functional groups, attenuated total reflectance-Fourier transform infrared spectroscopy (ATR-FTIR, Bruker Alpha II Compact, Karlsruhe, Germany) was employed using a diamond ATR crystal. For each sample, 32 spectral scans were collected with a resolution of 2 cm^−1^ in the range between 4000 cm^−1^ and 400 cm^−1^. Thermal behaviors of the MOF and membrane samples were investigated by heating the specimens in air to 800 °C at a ramp rate of 10 °C/min using a thermogravimetric analyzer (TGA, NETZSCH STA 449 F3 Jupiter, Selb, Germany).

### 2.5. Membrane Performance Analysis

Gas separation performance of the membranes was evaluated using an in-house built high throughput gas separation setup (HTGS), described elsewhere [38]. Measurements were conducted in triplicate at 35 °C and 3 bar with C_2_H_4_, C_2_H_6_, C_3_H_6_, C_3_H_8_ and an equimolar mixture of C_2_ or C_3_ olefin/paraffin gases. The constant-volume varying-pressure method was used to determine the gas permeability values of the membranes for single- and binary-component measurements. The time-dependent pressure change (dp/dt, cmHg/s) in a fixed-volume (V, cm^3^) auxiliary downstream cylinder was recorded using a pressure transducer (Baratron^®^, MKS, Munich, Germany), and the permeability of component i ($P_{i}$, Barrer) was calculated using Equation (1) for a known membrane thickness (L, cm), permeation area (A, cm^2^) and mole fraction of the component in the permeate and feed streams ($y_{i}$ and $x_{i}$, respectively) at a given temperature (*T*, K) and pressure (p, cmHg), where R represents the gas constant.

(1) $$P_{i}=\frac{10^{10}\times V\times L\times y_{i}}{p\times A\times R\times T\times x_{i}}\times \frac{dp}{dt}$$

Ideal C_2_H_4_/C_2_H_6_ and C_3_H_6_/C_3_H_8_ selectivities ($\alpha_{i/j}$) were calculated based on the ratio of the gas permeability values obtained in single-component measurements carried out with respective gases, while separation factors ($\alpha_{i/j}^{*}$) were determined by analyzing the permeate composition obtained from measurements performed with binary gas mixtures by a gas chromatograph (CompactGC, Interscience) using Equation (2).

(2) $$\alpha_{i/j}^{*}=\frac{y_{i}/y_{j}}{x_{i}/x_{j}}$$

where i and j indicate the olefin and paraffin components of the binary mixtures, respectively, while x and y represent the mole fractions of these components in the feed and permeate streams, respectively.

## 3. Results and Discussion

### 3.1. Characterization of MOF-808 Samples

**Figure 1:**
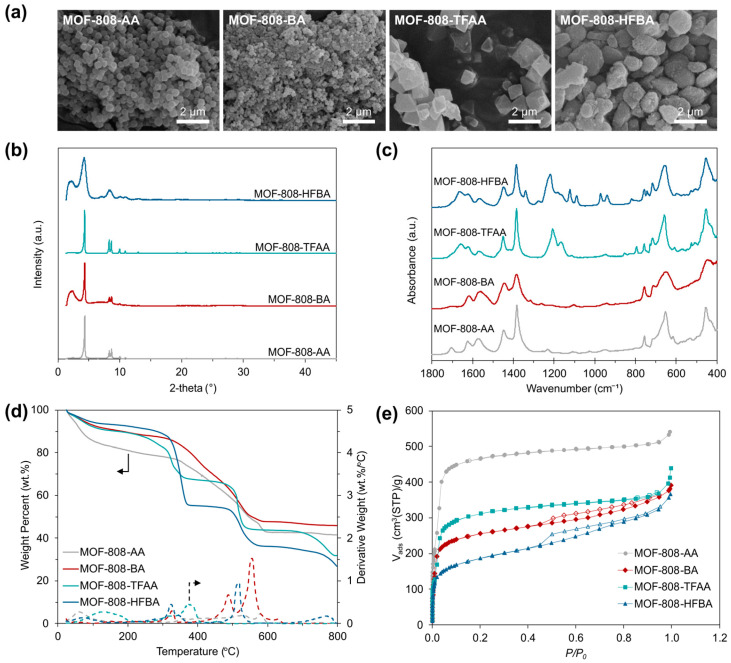
(**a**) SEM images, (**b**) XRD patterns, (**c**) FTIR spectra, (**d**) thermograms and (**e**) N_2_ sorption isotherms (where closed and open symbols represent adsorption and desorption branches, respectively) of MOF-808 samples synthesized with different modulators.

MOF-808 samples were synthesized using four different monoprotic carboxylic acid modulators (AA, BA, TFAA and HFBA) in order to investigate their potential as crystalline porous fillers in preparation of MMMs for C_2_H_4_/C_2_H_6_ and C_3_H_6_/C_3_H_8_ gas separation. The synthesized MOF samples were denoted according to corresponding modulators used during their syntheses (e.g., MOF-808-AA, MOF-808-BA, MOF-808-TFAA and MOF-808-HFBA) for clarity. After synthesis, the samples were first analyzed by various characterization methods to assess their morphological, structural and textural properties, as presented in Figure 1.

SEM images indicated an alteration in particle size and morphology depending on the modulator used for MOF-808 synthesis (Figure 1a), in accordance with other studies in the literature [34,39,40]. Zirconium-carboxylate clusters, serving as the building blocks of MOFs, are formed by coordinating deprotonated carboxylate groups to zirconium ions [41,42]. Competition for the binding sites of the zirconium cluster between the monocarboxylic acid modulators and the tricarboxylic acid linker greatly influences MOF morphology by regulating crystal nucleation and growth, depending on the size, acidity and hydrophilicity of the modulator used [34,42,43]. Modulators AA and BA resulted in samples with a smaller particle size compared to their fluorinated counterparts, TFAA and HFBA. Particles in spherical and octahedral shape with homogeneous pore size distributions were obtained when AA and TFAA were used as modulators for MOF-808 synthesis, respectively, while the use of BA and HFBA led to agglomerated particles with a lack of uniformity in size and shape. The increase in alkyl chain length, i.e., from AA and TFAA to BA and HFBA, respectively, appears to have caused a decrease in particle size, which could be attributed to steric hinderance resulting from the use of larger modulators.

XRD patterns presented in Figure 1b also support this. The crystallinity of MOF particles synthesized with different modulators was preserved, whereas a peak broadening was observed when BA and HFBA were used as modulators, indicating smaller crystallite formation due to long alkyl chains of BA and HFBA molecules [34].

Figure 1c compares the IR spectra of MOF-808 samples modulated with non-fluorinated and fluorinated counterparts. For all samples, the characteristic IR bands of the BTC linker were observed around 1620 cm^−1^, 1570 cm^−1^ and 1380 cm^−1^, while the bands attributed to (Zr–O) were detected around 650 cm^−1^ and 450 cm^−1^ [37,44]. The newly appeared doublet between 1200 cm^−1^ and 1100 cm^−1^ in the IR spectra of MOF-808-TFAA and MOF-808-HFBA was attributed to (C–F) stretching vibrations of (–CF_3_) groups [45], while an extra doublet observed around 1100 cm^−1^ in the spectra of MOF-808-HFBA could be denoted to (C–F) vibrations of aliphatic (–CF_2_) groups [46], confirming the presence of perfluoro compounds in these MOF samples.

The thermograms of the MOF samples indicated a three-step weight loss, as can be seen in Figure 1d. The initial weight loss below 100 °C can be attributed to the removal of adsorbed water molecules, while the second weight loss step observed up to 400 °C was ascribed to the removal of modulators and dehydroxylation of the metal nodes [47]. Finally, the collapse and decomposition of the framework was observed between 400 °C and 650 °C.

N_2_ physisorption data presented in Figure 1e revealed a reduced BET surface area and pore volume in the order of MOF-808-AA > MOF-808-TFAA > MOF-808-BA > MOF-808-HFBA, as listed in Table 1. Evidently, the use of longer (perfluoro)alkyl chains as modulators for MOF synthesis led to a lower surface area and pore volume. On the other hand, TFAA and HFBA resulted in a 32% and 26% lower surface area and 46% and 54% reduced pore volume, respectively, compared to their non-fluorinated counterparts. Despite having the same chain length, the lower values obtained here with more acidic modulators are attributed to the higher probability of modulator occupancy in the framework due to their higher degree of deprotonation [34].

### 3.2. Characterization of MOF-808/6FDD MMMs

**Figure 2:**
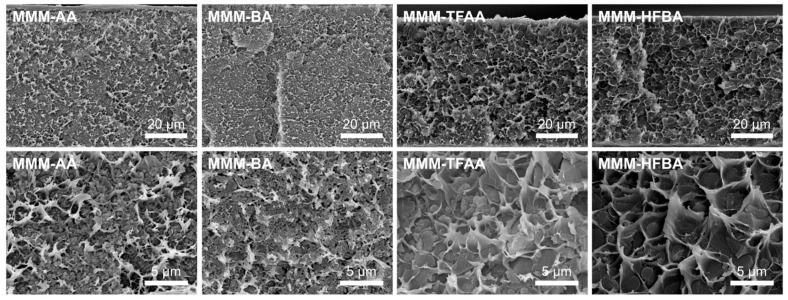
Cross-sectional SEM images of MOF-808/6FDD MMMs containing MOF-808 samples synthesized with different carboxylic acid modulators as filler at a magnification of 1000× (**top**) and 5000× (**bottom**).

After successful synthesis of MOF samples with different modulators, these particles, MOF-808-AA, MOF-808-BA, MOF-808-TFAA and MOF-808-HFBA, were incorporated into 6FDD membranes to prepare MOF-808/6FDD MMMs, denoted as MMM-AA, MMM-BA, MMM-TFAA and MMM-HFBA, respectively. The cross-sectional SEM images shown in Figure 2 indicate a uniform incorporation of the MOF particles into the 6FDD polymer matrix and the formation of crater-like patterns due to the stress at filler–polymer interfaces [37]. No particle agglomerates or clusters that could cause interfacial defects between the fillers and polymer matrix were observed, suggesting a good compatibility between 6FDD and well-dispersed MOF-808 particles [37].

**Figure 3:**
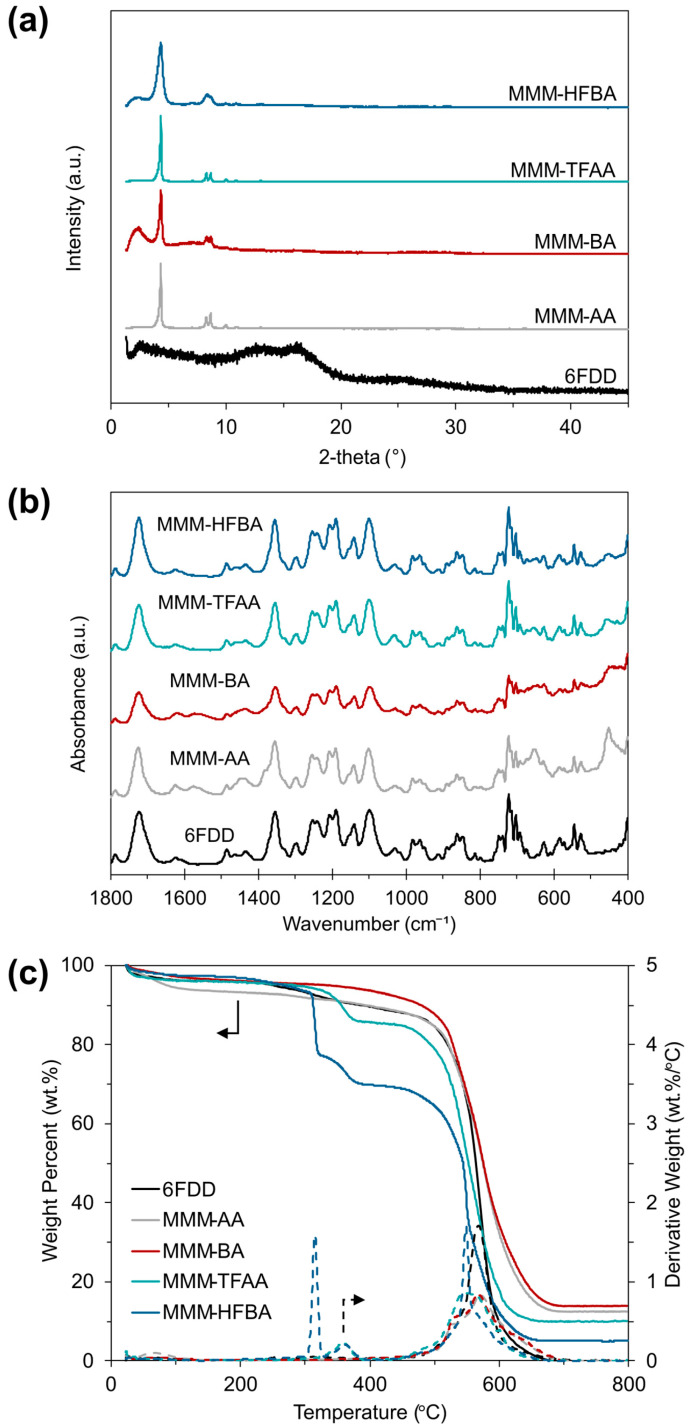
(**a**) XRD patterns, (**b**) ATR-FTIR spectra and (**c**) thermograms of unfilled 6FDD and MOF-808/6FDD membranes.

The diffractogram of the unfilled 6FDD membrane in Figure 3a shows a broad peak between 10° and 20° due to its amorphous nature [48], while the crystalline MOF particles dominate the XRD patterns of the MMMs, without showing a change in peak positions of corresponding incorporated MOFs, indicating a well preserved crystallinity of the particles in these MMMs.

IR spectra of the unfilled and MOF-808-incorporated membranes are presented in Figure 3b. In the spectra of all membranes, the characteristic bands attributed to (C=O) and (C–N) stretching vibrations were observed at 1720 cm^−1^ and 1360 cm^−1^, respectively [49]. On the other hand, the peaks located in the low wavenumber region between 500 cm^−1^ and 400 cm^−1^ in the spectra of the MMMs, which corresponds to (Zr–µ_3_–OH) stretching vibrations of the MOFs [37], confirm the successful incorporation of the filler particles into the polymer matrix. Thermograms of the unfilled 6FDD and MMMs shown in Figure 3c demonstrate that the membranes are thermally stable up to 300 °C, where the thermal decomposition of the MOF samples begins to occur (Figure 1d). With increasing temperature after this point, MMM-TFAA and MMM-HFBA experienced a more pronounced weight loss between 300 °C and 400 °C compared to MMM-AA and MMM-BA, which might be attributed to the facilitation of decomposition by fluorination [50]. Nonetheless, the beginning of the main thermal degradation of the polymer backbone was not affected by filler incorporation and was observed after 490 °C for all membrane samples.

### 3.3. C_2_ and C_3_ Olefin/Paraffin Gas Separation Performance of MOF-808/6FDD MMMs

**Figure 4:**
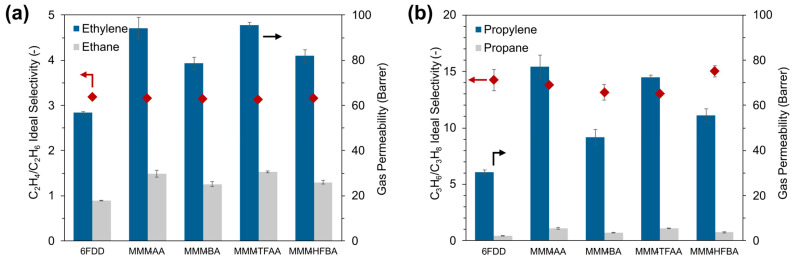
Performance of unfilled 6FDD membrane and MMMs containing MOF-808 samples synthesized with different modulators for (**a**) C_2_H_4_/C_2_H_6_ and (**b**) C_3_H_6_/C_3_H_8_ gas separation. Measurements were conducted at 3 bar and 35 °C using single-component feed streams.

Gas permeation measurements were carried out on the unfilled 6FDD membrane and the MOF-808/6FDD MMMs for C_2_H_4_, C_2_H_6_, C_3_H_6_ and C_3_H_8_ gases, as depicted in Figure 4. For C_2_ separation, the unfilled 6FDD membrane showed an ideal C_2_H_4_/C_2_H_6_ selectivity of 3.2 with corresponding permeability values of 57 Barrer and 18 Barrer for C_2_H_4_ and C_2_H_6_, respectively. Upon incorporation of the particles, MMMs exhibited a substantial increase in gas permeability for both C_2_H_4_ and C_2_H_6_. The improvement in gas permeability was more pronounced for the MMMs containing MOFs with shorter alkyl chains, i.e., MMM-AA and MMM-TFAA, compared to those with longer chains, i.e., MMM-BA and MMM-HFBA (Figure 4a), which can be related to the accessible pore volume within the MOF pores (Table 1). However, no significant difference was observed between the performance of MMMs containing MOFs synthesized with non-fluorinated and fluorinated counterparts of the carboxylic acids with the same alkyl chain length. For both MMM-AA and MMM-TFAA, the C_2_H_4_ permeability increased by 67% compared to that of the unfilled 6FDD, reaching 95 Barrer, while MMM-BA and MMM-HFBA led to a C_2_H_4_ permeability of 80 Barrer with a 40% improvement. Nevertheless, an increase in C_2_H_6_ permeability similar to that of C_2_H_4_ was also observed for all corresponding MMMs prepared, thus the improvement in gas permeability provided no enhancement in membrane selectivity. In brief, the incorporated MOF particles enhanced the diffusion of gas molecules but were unable to differentiate between C_2_H_4_ and C_2_H_6_ due to their relatively large pore sizes (Table 1 and Figure S2).

The measurements performed with C_3_H_6_ and C_3_H_8_ single gases led to an outcome similar to that observed for C_2_ separation. Gas permeability was enhanced upon filler incorporation, while the ideal selectivity exhibited by the unfilled 6FDD membrane was maintained with minor changes (Figure 4b). MMMs containing MOFs modulated with short-chain carboxylic acids (i.e., MMM-AA and MMM-TFAA) showed similar improvements in gas permeability, leading to values 2.5-times higher than that of the unfilled 6FDD membrane. On the other hand, the permeability increased by 54% and 84% for MMM-BA and MMM-HFBA, respectively, indicating limited improvement due to the pore space occupied by the long-chain modulators of the incorporated MOF particles, in line with the lower surface area and smaller pore volume of these samples determined from N_2_ physisorption measurements (Table 1). Here, the improvement in gas permeability became more prominent compared to C_2_ separation, suggesting that the introduced porosity with the incorporation of the fillers plays a greater role in contributing to the diffusivity of larger C_3_ molecules compared to C_2_ gases, whose diffusion is relatively less restricted by the polymer phase.

**Figure 5:**
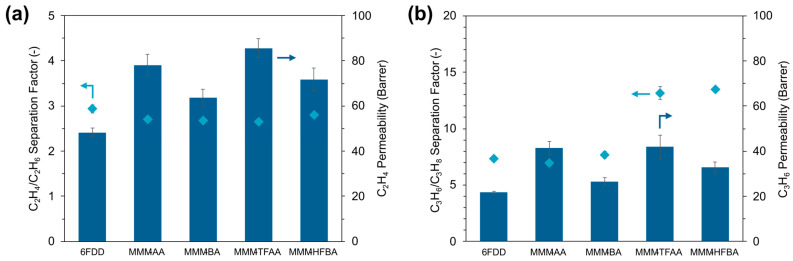
Mixed gas separation performance of the unfilled 6FDD membrane and MMMs containing MOF-808 samples prepared with different modulators for an equimolar mixture of (**a**) C_2_H_4_/C_2_H_6_ and (**b**) C_3_H_6_/C_3_H_8_ gases. Measurements were carried out at 3 bar and 35 °C.

Since the co-presence of these gases can drastically affect the separation performance if one gas preferentially interacts with the membrane over the other, permeation experiments were performed under mixed gas conditions to more realistically evaluate the membrane performance (Figure 5). Similar to the single gas measurements, MMMs provided an increase in gas permeability for the equimolar mixture of C_2_H_4_ and C_2_H_6_ gases while exhibiting a similar separation factor compared to the unfilled 6FDD (Figure 5a), implying that the fluorination of the MOF-808 samples had no influence on the overall C_2_H_4_/C_2_H_6_ selectivity of the membranes. Sun et al. showed that the fluorinated MOF-808 samples prepared by post-synthetic modification exhibited greater affinity for C_2_H_6_ compared to C_2_H_4_ due to hydrogen-bonding and van der Waals interactions [51]. Hence, the MMMs prepared by incorporating these fluorinated MOFs into PIM-1 led to a C_2_H_6_-selective permeation. Considering the ultra-high permeability of the PIM-1 membrane due to inefficient molecular packing [52], it can be interpreted that the strong adsorption affinity of the embedded MOFs towards C_2_H_6_ contributed more significantly to MMM performance, leveraging membrane selectivity for C_2_H_6_ over C_2_H_4_. For the 6FDD-based MMMs investigated in this study, however, the membrane selectivity appeared to be mainly governed by the diffusion characteristics of 6FDD, which precluded the exploitation of the C_2_H_6_ affinity of the fluorinated pore environment of MOF-808 particles.

For C_3_ separation, a divergence was noticeable between the performance of the MMMs containing MOFs with non-fluorinated and perfluorinated modulators (Figure 5b). On the one hand, the C_3_H_6_ permeability almost doubled upon incorporation of MOF-808-AA into 6FDD, while this improvement was only 22% when MOF-808-BA was incorporated. Considering that the separation factors remained constant for MMM-AA and MMM-BA, this could be an indication that the confined pore environment of MOF-808-BA hindered gas diffusion compared to MOF-808-AA, limiting the improvement in gas permeability of the resulting MMM. However, the gas–framework interactions in these MOFs did not prevail upon membrane selectivity. On the other hand, incorporating MOF-808-TFAA and MOF-808-HFBA into 6FDD improved the mixed gas C_3_H_6_/C_3_H_8_ separation factor, along with the enhanced C_3_H_6_ permeability, leading to a promising MMM performance comparable to the 6FDA-based MMMs reported for C_3_ separation in the literature [53,54,55]. With both MOFs, the C_3_H_6_/C_3_H_8_ separation factors of the corresponding MMMs almost doubled compared to the unfilled 6FDD membrane. The C_3_H_6_ permeability of MMM-TFAA and MMM-HFBA reached 42 Barrer and 33 Barrer, respectively, which were similar to the improvements attained by their non-fluorinated counterparts. Additionally, the improved C_3_ separation performance by incorporating fluorinated MOF-808 was observed to be maintained under continuous operation for 72 h (Figure S3).

Despite the similar permeability enhancement observed for both non-fluorinated and fluorinated counterparts, the improvement in separation factors achieved with MMMs containing fluorinated MOFs can be attributed to preferential host–guest interactions between the MOF and C_3_ gases. The fluorinated pore environment of MOF-808-TFAA and MOF-808-HFBA can induce better hydrogen-bonding interactions with C_3_H_8_ molecules compared to C_3_H_6_ [51,56,57], slowing down their diffusion through the MOF-808 pores. This change in diffusivity could possibly be the reason for the limited enhancement in C_3_H_8_ permeability of the corresponding MMMs, resulting in higher C_3_H_6_/C_3_H_8_ separation factors compared to the unfilled 6FDD membrane. A similar occurrence was also reported for UTSA-280 incorporated MMMs [58]. UTSA-280 showed an excellent adsorption selectivity for C_2_H_4_/C_2_H_6_ separation owing to its C_2_H_4_-selective sieving over C_2_H_6_ [59]. However, incorporation of UTSA-280 into the more permeable 6FDA-DAM resulted in lower C_2_H_4_ permeability than that of the unfilled membrane without affecting membrane selectivity; while when the less permeable 6FDA-DAM:DABA (3:2) was used as the polymer matrix for MMM preparation, 32% higher C_2_H_4_/C_2_H_6_ selectivity and 15% improved C_2_H_4_ permeability were obtained compared to the unfilled membrane [58]. Considering the hybrid nature of MMMs, the prominence of permeability matching of polymer-filler pairs should be taken into account for the successful MMM development, as the final membrane performance is not only affected by the incorporated filler particles but also controlled by the solubility and diffusivity characteristics of the polymer matrix used.

### 3.4. Effect of Temperature on Olefin/Paraffin Separation Performance

**Figure 6:**
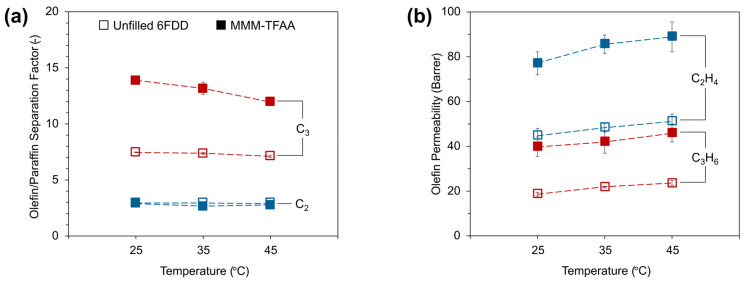
Effect of operating temperature on (**a**) olefin/paraffin separation factor and (**b**) olefin permeability of unfilled 6FDD and MMM-TFAA for C_2_H_4_/C_2_H_6_ (C_2_) and C_3_H_6_/C_3_H_8_ (C_3_) separation. Measurements were performed at 3 bar and 35 °C using equimolar gas mixtures.

To better understand the permeation properties of these membranes, measurements were performed at different temperatures for equimolar C_2_H_4_/C_2_H_6_ and C_3_H_6_/C_3_H_8_ mixtures (Figure 6). For both unfilled 6FDD and MMM-TFAA, temperature appeared to have no significant impact on the C_2_H_4_/C_2_H_6_ separation factor (Figure 6a), while a 15% increase in C_2_H_4_ permeability was observed when the temperature was increased from 25 °C to 45 °C for both membranes (Figure 6b).

This trend between temperature and permeability is also evident for C_3_ separation. Although the sorption of these condensable gases decreases with elevating temperature [60], the counter-effect of increased diffusivity appears to play a more decisive role in determining the final permeability of these membranes. For the unfilled 6FDD membrane, a 26% increase in C_3_H_6_ permeability was observed upon temperature rise from 25 °C to 45 °C, without a significant effect on the C_3_H_6_/C_3_H_8_ separation factor. On the other hand, even though the increase in C_3_H_6_ permeability was only 15% for MMM-TFAA, this increase was accompanied by a concomitant decrease in C_3_H_6_/C_3_H_8_ separation factor from 13.9 to 11.9. Considering that the separation factor of the unfilled 6FDD membrane remains stable in this temperature range, it can be inferred that the response of the polymer phase to temperature change is similar for C_3_H_6_ and C_3_H_8_ permeabilities, alluding to altered interactions between MOF-808-TFAA and C_3_ gases. Thus, this decrease in the C_3_H_6_/C_3_H_8_ separation factor of MMM-TFAA can possibly be ascribed to the noticeable increase in C_3_H_8_ permeability due to the weakening of host–guest interactions between MOF and C_3_H_8_ with increasing temperature [51].

## 4. Conclusions

Four isostructural MOF-808 samples were synthesized using different carboxylic acid modulators. The synthesized MOFs were incorporated as fillers into 6FDD to assess the effect of chain length and fluorination of the MOF modulator on the C_2_ and C_3_ olefin/paraffin separation performance of the resulting MMMs. Incorporation of all MOF particles resulted in increased permeability for each gas examined, while the ideal C_2_H_4_/C_2_H_6_ and C_3_H_6_/C_3_H_8_ selectivities of these MMMs remained constant compared to the unfilled 6FDD membrane for these single gas measurements. The improvement in gas permeability was found to be inversely correlated with the alkyl chain length of the modulator, while fluorination on modulator showed no significant impact on MMM permeability. The effect of the co-presence of olefins and paraffins on membrane performance was investigated using equimolar C_2_H_4_/C_2_H_6_ and C_3_H_6_/C_3_H_8_ mixtures. For C_2_ separation, the results indicated a trend similar to that obtained in single gas measurements. The temperature dependency of the C_2_ separation performance of the MMMs appeared to be largely dependent on the polymer phase response. With increasing temperature, an increase in C_2_H_4_ permeability was observed in both unfilled 6FDD and MMM-TFAA without changing the C_2_H_4_/C_2_H_6_ separation factors. On the other hand, a remarkable improvement in the C_3_H_6_/C_3_H_8_ separation factor was observed upon incorporation of fluorinated MOFs under mixed gas conditions, along with an increase in C_3_H_6_ permeability. MMM-TFAA and MMM-HFBA led to 79% and 84% higher C_3_H_6_/C_3_H_8_ separation factors, while C_3_H_6_ permeability was increased by 93% and 52%, respectively, compared to the unfilled 6FDD membrane. The C_3_H_6_/C_3_H_8_ separation factor remained stable for the unfilled 6FDD membrane, while a decrease was observed for MMM-TFAA due to the increased C_3_H_8_ permeability with increasing temperature. As demonstrated here for MOF-808/6FDD MMMs, the separation performance of the resulting MMM strongly relies on the selectivity/permeability match of its components. Thus, meticulous selection of the polymer matrix and filler particles is vital for the success of developing MMMs with enhanced separation performance for any target gas mixture.

## Figures and Tables

**Table 1:** Surface area, pore volume and average pore size of MOF-808 samples.

Sample	Surface Area * (m^2^/g)	Pore Volume (cm^3^/g)	Pore Size (Å)
MOF-808-AA	1518	0.54	16.6
MOF-808-BA	846	0.24	14.8
MOF-808-TFAA	1031	0.29	16.6
MOF-808-HFBA	637	0.11	13.1

* Based on the Brunauer–Emmett–Teller (BET) theory.

## Supplementary Materials

The following supporting information can be downloaded at: https://www.mdpi.com/article/10.3390/membranes15100290/s1, Figure S1: Chemical structures of (a) 6FDD and (b) modulators used in MOF-808 synthesis. Figure S2: Pore size distribution of MOF-808 samples modulated with different chain length (a) alkyl carboxylic acids (acetic acid (AA) and butyric acid (BA)) and (b) perfluoroalkyl carboxylic acids (trifluoroacetic acid (TFAA) and heptafluorobutyric acid (HFBA)). Figure S3: Operational stability of MMM-TFAA for C_3_H_6_/C_3_H_8_ separation over 72 h.

## Abbreviations

The following abbreviations are used in this manuscript:

6FDA	4,4′-(Hexafluoroisopropylidene)diphthalic anhydride
DAM	2,4-Diaminomesitylene
DABA	3,5-Diaminobenzoic acid
