# Supplementary material for: 6FDA-Based Co-Polyimide Membranes Incorporating Modulated MOF-808s for Olefin/Paraffin Gas Separations
Source: Membranes (Basel). 2025 Sep 25;15(10):290. doi: 10.3390/membranes15100290 (PMC12566319; doi:10.3390/membranes15100290)
Supplement: Supplementary file 1 [file membranes-15-00290-s001.zip › membranes-3829787-supplementary.pdf]

Supplementary Information: "6FDA-Based Co-Polyimide Membranes Incorporating Modulated MOF-808s for Olefin/Paraffin Gas Separations"

Harun Kulak, Lore Hannes and Ivo F. J. Vankelecom *

Membrane Technology Group (MTG), Centre for Membrane Separations, Adsorption, Catalysis and Spectroscopy for Sustainable Solutions (cMACS), Faculty of Bioscience Engineering, KU Leuven, Celestijnenlaan 200F, P.O. Box 2454, 3001 Leuven, Belgium
* Correspondence: ivo.vankelecom@kuleuven.be

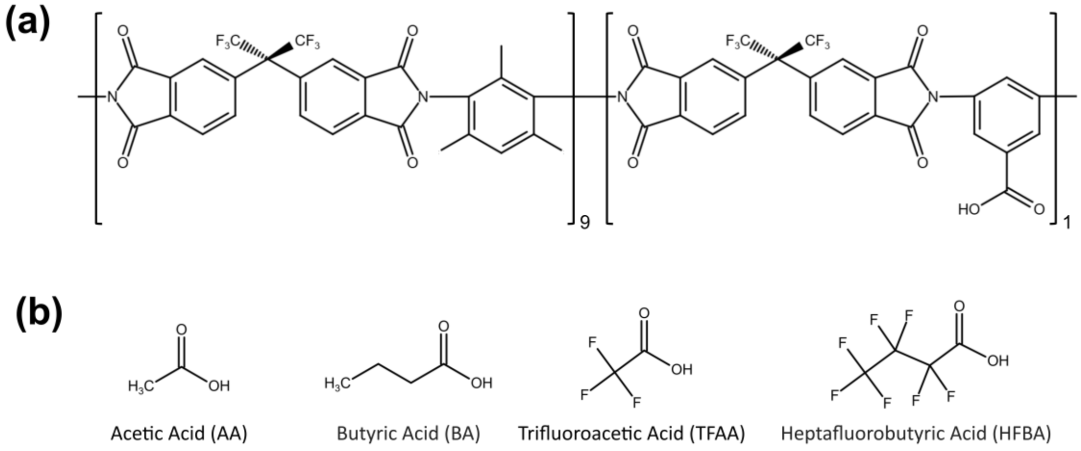

Figure S1. *Chemical structures of **(a)** 6FDD, and **(b)** modulators used in MOF-808 synthesis.*

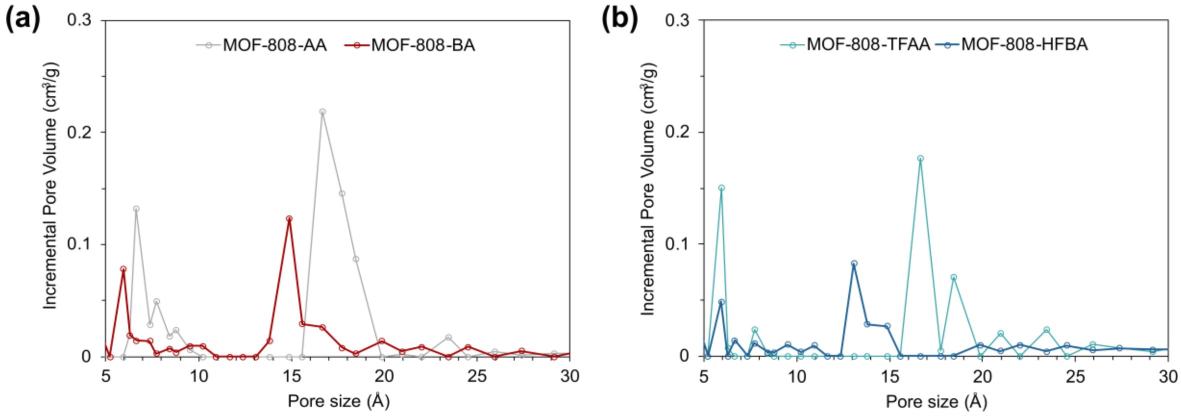

Figure S2. *Pore size distribution of MOF-808 samples modulated with different chain length **(a)** alkyl carboxylic acids (acetic acid (AA) and butyric acid (BA)) and **(b)** perfluoroalkyl carboxylic acids (trifluoroacetic acid (TFAA) and heptafluorobutyric acid (HFBA)).*

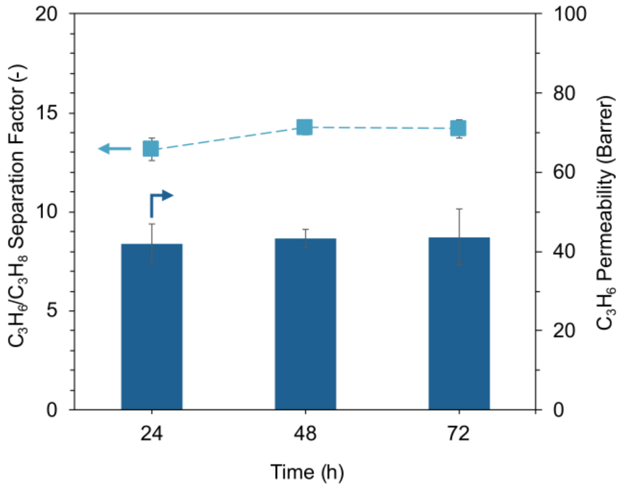

Figure S3. *Operational stability of MMM-TFAA for C_3H_6/C_3H_8 separation over 72 h. Measurements were carried out using an equimolar gas mixture at 3 bar and 35 °C.*
